# Mixed Reality in Undergraduate Nursing Education: A Systematic Review and Meta-Analysis of Benefits and Challenges

**DOI:** 10.3390/nursrep15050137

**Published:** 2025-04-22

**Authors:** Laura Guillen-Aguinaga, Esperanza Rayón-Valpuesta, Sara Guillen-Aguinaga, Blanca Rodriguez-Diaz, Rocio Montejo, Rosa Alas-Brun, Enrique Aguinaga-Ontoso, Luc Onambele, Miriam Guillen-Aguinaga, Francisco Guillen-Grima, Ines Aguinaga-Ontoso

**Affiliations:** 1 Department of Nursing, Kystad Helse-og Velferdssenter, 7026 Trondheim, Norway; lguillen@alumni.unav.es; 2 Department of Nursing, Clinica Universidad de Navarra, 28027 Madrid, Spain; 3 Department of Nursing, Complutense University of Madrid, 28040 Madrid, Spain; erayon@ucm.es; 4 Department of Health Sciences, Public University of Navarra, 31008 Pamplona, Spain; sguillen.4@alumni.unav.es (S.G.-A.); ines.aguinaga@unavarra.es (I.A.-O.); 5 Peralta Health Care Center, Navarra Health Service, 31350 Peralta, Spain; 6 Department of Preventive Medicine, Clinica Universidad de Navarra, 28027 Madrid, Spain; brodriguezdi@unav.es; 7 Department of Obstetrics and Gynecology, Institute of Clinical Sciences, University of Gothenburg, 413 46 Gothenburg, Sweden; rocio.montejo.rodriguez@gu.se; 8 Department of Obstetrics and Gynecology, Sahlgrenska University Hospital, 413 46 Gothenburg, Sweden; 9 Department of Sociosanitary Sciences, University of Murcia, 30120 Murcia, Spain; aguinaga@um.es; 10 Department of Preventive Medicine, Virgen de la Arrixaca University Clinical Hospital, 30003 Murcia, Spain; 11 School of Health Sciences, Catholic University of Central Africa, Yaoundé 1100, Cameroon; onambele.luc@ess-ucac.org; 12 School of Law, International University of La Rioja, 26006 Logroño, Spain; miriamguillenaguinaga@gmail.com; 13 Group of Clinical Epidemiology, Area of Epidemiology and Public Health, Healthcare Research Institute of Navarre (IdiSNA), 31008 Pamplona, Spain; 14 CIBER in Epidemiology and Public Health (CIBERESP), Institute of Health Carlos III, 46980 Madrid, Spain; 15 Department of Preventive Medicine, Clinica Universidad de Navarra, 31008 Pamplona, Spain

**Keywords:** mixed reality, augmented reality, virtual reality, nursing education, nursing education research, systematic review, meta-analysis

## Abstract

**Background:** Nursing Schools are incorporating Mixed Reality (MR) into student training to enable them to confront challenging or infrequently encountered scenarios in their practice and ensure their preparedness. This systematic review evaluates the benefits and challenges of implementing MR in nursing curricula. **Materials and Methods:** A search was conducted in PubMed, WOS, Scopus, Embase, and CINAHL for studies published between 2011 and 2023. The search strategy used was “(nurses OR nurse OR nursing) AND mixed reality AND simulation”. Inclusion criteria required that studies focus on undergraduate nursing students and be written in English or Spanish. Exclusion criteria included reviews, bibliometric studies, and articles that did not separately report undergraduate nursing student results. Quality was evaluated with the JBI Critical Appraisal Checklist for Qualitative Research and the Newcastle-Ottawa Scale. A meta-analysis was conducted on studies with control groups to compare MR’s effectiveness against traditional teaching methods. **Results:** Thirty-three studies met the inclusion criteria. MR was widely used to improve clinical judgment, patient safety, technical skill acquisition, and student confidence. The meta-analysis found that MR reduced anxiety (Cohen’s d = −0.73, *p* < 0.001). However, its impact on knowledge acquisition and skill development was inconsistent. There was no improvement over traditional methods (*p* = 0.466 and *p* = 0.840). Despite positive qualitative findings, methodological variability, small sample sizes, and publication bias contributed to mixed quantitative results. The main challenges were cybersickness, usability, high costs, and limited institutional access to MR technology. **Conclusions:** Although MR can help nursing education by decreasing students’ anxiety, its efficacy remains inconclusive. Future research should use larger, randomized controlled trials to validate MR’s role in nursing education.

## 1. Introduction

Nursing education influences patient survival rates and clinical outcomes [1,2]. Nurses work in dynamic and unpredictable environments where they must assess and respond to patient needs [3,4].

Conventional methods, such as lectures and low-fidelity simulations (mannequins, case studies, and role-playing), often lack the realism required for effective skill acquisition [3,5,6,7,8,9]. Immersive technologies such as AR and VR have also proven particularly useful in training emergency and disaster response competencies, where realism and autonomous practice are crucial. For example, Fant (2025) highlights their effective use by emergency agencies and their positive impact on nursing students’ engagement and confidence during disaster preparedness training [10]. Fidelity, or the degree of realism in a simulation, is a key factor in learning outcomes. Mannequins do not provide sufficient realism for effective nursing education [11]. High-fidelity Virtual Reality simulations immerse learners in computer-generated clinical environments, enabling realistic interaction through standardized scenarios [12,13,14,15]. According to the INACSL, VR is “a computer-generated reality, which allows learners to experience various auditory and visual stimuli through specialized ear and eyewear, such as head-mounted displays” (HMDs) [16].

Augmented Reality (AR) overlays digital elements in real-world environments, enhancing clinical training with interactive visuals and real-time data [17,18]. Mixed Reality (MR) combines the visual enhancements of Augmented Reality (AR) with the immersive features of Virtual Reality (VR), enabling dynamic, bidirectional interaction between virtual and physical elements to increase simulation realism [19].

**Figure 1:**
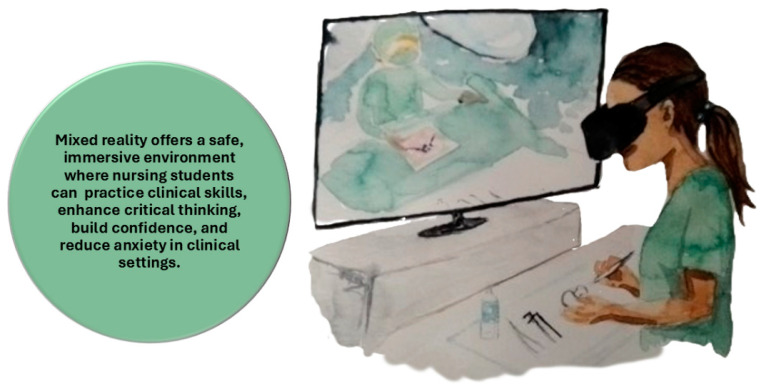
Watercolor illustration by Sara Guillen-Aguinaga: nursing student performing a procedure on a patient simulator using augmented reality goggles.

MR, along with standardized patients and professional actors, has been increasingly incorporated into nursing curricula to provide a realistic and interactive learning environment where students can practice without risking patient safety, especially for early-year students who possess theoretical knowledge but lack practical experience—an issue also reported by many students during their transition into professional clinical roles [20,21,22,23]. These technologies fall under the broader category of Extended Reality (XR). This umbrella term encompasses VR, AR, and MR and refers to all immersive digital environments that enhance or replace the physical world to support interactive learning experiences [24]. Many students reported feeling inadequately prepared for clinical responsibilities [25,26,27,28]. The application of AR in clinical training is illustrated in Figure 1, where a nursing student utilizes Augmented Reality goggles while performing a procedure on a patient simulator.

AR, MR, and VR have led to immersive simulations (ISs) where students are exposed to complex or rare clinical scenarios [29]. Immersion is a psychological response triggered by interaction with virtual environments that enhances learning by fostering a strong sense of presence and engagement [9]. The extent of immersion depends on how many sensory modalities are engaged; the more senses involved, the stronger the immersion effect [30]. A highly immersive experience is defined by these key dimensions: inclusivity, extensiveness, surround sound/visuals, vividness, and coherence, which enhance the user’s sense of presence [31,32,33]. A recent quasi-experimental study by Rodríguez-Abad et al. (2022) found that an online AR experience significantly improved nursing students’ academic performance in leg ulcer care, particularly in autonomous learning and 3D comprehension, compared to a traditional face-to-face AR experience [34].

MR nursing simulations promote skill development, critical thinking, and decision-making [35,36,37]. The difference between AR and MR is that AR overlays digital content onto real-world environments without enabling interaction, whereas MR allows seamless interaction between the two [38]. MR may enhance engagement and clinical judgment in nursing students, though evidence of its overall effectiveness remains mixed [3,39]. A recent study has demonstrated how Mixed Reality combined with bimanual haptic feedback can enhance IV insertion training by offering a more immersive and customizable clinical environment, including variable patient features such as vein size and skin stiffness, thereby improving simulation realism and adaptability [40]. Although over 1200 studies have examined VR in professional healthcare contexts between 2006 and 2022, relatively few have focused on its educational impact among nursing students. Evidence on the effectiveness of MR in this context remains mixed [41]

## 2. Materials and Methods

### 2.1. Research Question

The study aimed to analyze the use of MR in undergraduate nursing education, evaluating its impact on students’ knowledge, skills, engagement, and self-efficacy and the barriers to its integration into curricula.

The study formulated the research question using the PICO (Patient/Problem, Intervention, Comparison, and Outcome) criteria [42,43], which have been recommended for use in nursing [44]. The question’s purpose was to review and qualitatively analyze whether Mixed Reality (I) improves learning outcomes and satisfaction (O) of nursing students (P) compared to a simulation (C).

#### 2.1.1. Primary Research Questions

The primary research questions were as follows:

1. Does the implementation of MR in undergraduate nursing education improve nursing students’ learning outcomes compared to traditional simulation methods?
2. Does the implementation of MR enhance undergraduate nursing students’ satisfaction levels compared to traditional simulation methods?
3. What challenges and limitations do undergraduate nursing students face when learning through Mixed Reality compared to traditional simulation?

#### 2.1.2. Secondary Research Questions

The secondary research questions aimed to explore additional aspects of Mixed Reality implementation in nursing education:

1. How does using MR influence skill acquisition, student engagement, and self-efficacy compared to traditional simulation?
2. What are the pedagogical and technological barriers to implementing MR in undergraduate nursing curricula?
3. What are the long-term implications of MR for shaping the future of nursing education in an increasingly complex healthcare environment?

### 2.2. Design

The review followed the PRISMA Guidelines [45,46,47], and partially the Cochrane guidelines for rapid reviews, using a simplified data extraction strategy and limiting the number of databases searched to enhance timeliness while maintaining methodological rigor. We registered the protocol in PROSPERO under the number CRD42023445679 and uploaded it to the Open Science Framework of the Center for Open Science, where it is available at https://doi.org/10.17605/OSF.IO/WSKPM (accessed on 4 March 2025).

### 2.3. Exclusion and Inclusion Criteria

The articles had to meet the following criteria: original research conducted with undergraduate nursing students, written in English or Spanish. Reviews, bibliometric studies, editorials, and opinion articles were excluded. Articles, where undergraduate students participated alongside postgraduates or professionals were excluded if their results were not reported separately.

### 2.4. Search Strategy

The following search strategy was created in natural language: “(nurses OR nurse OR nursing) AND mixed reality AND simulation”. Five databases were used: PUBMED, WOS, Scopus, Embase, and CINHAL. The search was confined to the last 12 years (2011–2023) and conducted on 2 January 2024. Two authors (EAO and FGG) carried out the bibliographic search. The study analyzed publications from 2011 to 2023 because these years capture the rapid evolution and adoption of MR in nursing education. Before 2011, the academic literature on MR-based teaching strategies was sparse and often limited to conceptual or pilot studies. Limiting the search through 2023 allowed for a comprehensive analysis of the most current findings up to the point of the bibliographic search conducted in January 2024, recognizing that the subsequent drafting and review process continued until March 2025. A further bibliographic search was conducted on 10 April 2025, and relevant references have been added to this text.

### 2.5. Selection of Articles

After the literature search, duplicate studies were identified and removed using EndNote software (version 20, Clarivate Analytics, Philadelphia, PA, USA). The studies were selected using the Rayyan web platform [48] and its associated mobile application.

Two independent reviewers (LGA and FGG) screened the retrieved studies’ titles, abstracts, and keywords. In cases of discrepancies, a third reviewer (EA) intervened to resolve them through discussion with the initial reviewers.

The titles and abstracts of the articles were screened using the predefined eligibility criteria explained previously (Section 2.3). Studies meeting these criteria were retrieved for full-text evaluation. All authors reviewed the full text of eligible studies. Studies were transcribed into Excel, where relevant data were systematically collected.

### 2.6. Data Extraction from the Articles

After the initial search and thorough evaluation of all the articles, a team of four authors (SGA, FGG, LGA, and IAO) individually scrutinized the texts of all shortlisted studies to finalize their inclusion. We resolved any discrepancies observed in their choices through collective discussion. If we could not reach a consensus, we referred the matter to a fifth author (EAO) for arbitration. We selected the following information from all the articles: the database in which the reference was found (PUBMED, WOS, Scopus, Embase, and CINHAL), Title, Authors, Year of Publication, DOI, PMID (Pubmed ID number of the article in PubMed), and bibliographic citation. Pairs of authors, RAB-ER and BG-LO, extracted the information to be included in the tables. It was reviewed and synthesized by LGA. In this systematic review, we encountered studies employing diverse measurement units, including seconds, percentages of correct responses, and various scale scores. We calculated the relative increase for each outcome measure to enable meaningful comparisons across these heterogeneous metrics. This approach standardizes the effect sizes, facilitating a consistent comparison of intervention impacts across studies with differing units of measurement [49].

### 2.7. Quality Assessment

All the selected quantitative articles were evaluated using the Newcastle–Ottawa Quality Assessment Scale (NOS) [36] to assess the quality of the articles [50]. This scale, widely used in systematic reviews [51], was initially developed for cohort and case-control studies and later adapted for cross-sectional studies [52,53,54], where the Modesti adaptation was used [55,56]. The scale contemplates three dimensions: study selection, comparability, and verifying the exposure or outcome of interest. It uses a star-based rating system: up to two stars ✸✸ for comparability, three stars ✸✸✸ for exposure and outcome, and four stars ✸✸✸✸ for selection. The total number of stars was also calculated.

The quality of clinical trials was evaluated with the Risk of Bias 2 (RoB 2) [57,58], recommended by the Cochran Collaboration [59].

The JBI Critical Appraisal Checklist for Qualitative Research (JBICACQR) was used for qualitative studies and the qualitative components of mixed-method designs [60,61]. Discrepancies in appraisal scores were resolved through consensus between the reviewers, and when necessary, a third reviewer was consulted to reach a final decision

### 2.8. Statistical Analysis and Software

A meta-analysis of studies with a control group was conducted. The meta-analysis was conducted using IBM SPSS Statistics version 29. Meta-analyses were conducted for skills, knowledge, anxiety, and trust. For anxiety, one study contributed four separate measures. Similarly, in the meta-analysis of trust, one study assessed trust using three different measures. The measures were combined into a single estimate using the following formulas: pooled mean and standard deviation calculations.

(1) $$M{ean}_{pooled}=\frac{\sum {Mean}_{i}}{n}\;$$

(2) $${SD}_{pooled}=\sqrt{\frac{\sum \left({SD}_{i}^{2}+\;{\left({Mean}_{i}-{Mean}_{pooled}\right)}^{2}\;\right)\;\;\;\;\;}{n}}\;$$

Mean_i_ and SD_i_ represent each anxiety measure’s mean and standard deviation (SD), Mean_pooled_ represents the overall mean, and n is the number of measurements.

In cases with means but no SD in the publications, the SD was estimated from Student’s *t*-values using the following formula.

(3) $$SD=\frac{\left|M_{1}-M_{2}\right|}{t}\times \sqrt{\frac{n_{1}n_{2}}{n_{1}+n_{2}}}$$

M is the mean, t is Student’s t, and n is the group size.

SD was estimated using the boxplot data in studies where the standard deviation (SD) was not provided. Specifically, each group’s quartile values (Q1 and Q3) were extracted from the boxplot images, and the SD was calculated based on the interquartile range (IQR). The following formula was applied [62,63]:

(4) $$SD\approx \frac{IQR}{1.35}$$

where IQR = Q3 − Q1, assuming an approximately normal distribution.

In those studies where only the mean and the range were available, SD was estimated using the following formula [64,65].

(5) $$SD\approx \frac{range}{6}$$

In those studies where the only data were the mean and the student t for paired data, the SD was estimated using the formula provided below [66,67]:

(6) $$SD=\frac{diference\;of\;means\;\;}{t}\times \sqrt{n}$$

where n is the number of pairs and t is the paired *t*-statistic

In studies where two different interventions were compared against the same control group, we treated each comparison as an independent study to be included in the meta-analysis. However, to account for the shared control group and to avoid overestimating its influence, we divided the sample size of the control group equally between the two comparisons while maintaining the original mean and standard deviation.

Heterogeneity was assessed using I^2^, Tau^2^, and Cochran’s Q statistics to determine the most appropriate model (fixed or random effects). We generated funnel plots and conducted Egger’s regression to assess potential bias. Although funnel plots visually inspect asymmetries in study results, Egger’s test provides a quantitative measure to detect small-study effects.

The descriptive statistical analysis was computed with IBM SPSS v29. We computed JoinPoint regression, using the program JoinPoint from the NCI, to study the trends in the number of papers. Maps were generated using the Mapchart software (version 4.3.2) [68]. The color schemes utilized in the maps were designed with Colorbrewer (v.2.0) [69,70]. In cases where numerical data were not explicitly reported in the articles, we extracted quantitative values from published graphs using PlotDigitizer software version 3 (https://plotdigitizer.com/app, accessed on 4 March 2025) [71].

## 3. Results

### 3.1. Study Selection and Distribution

**Figure 2:**
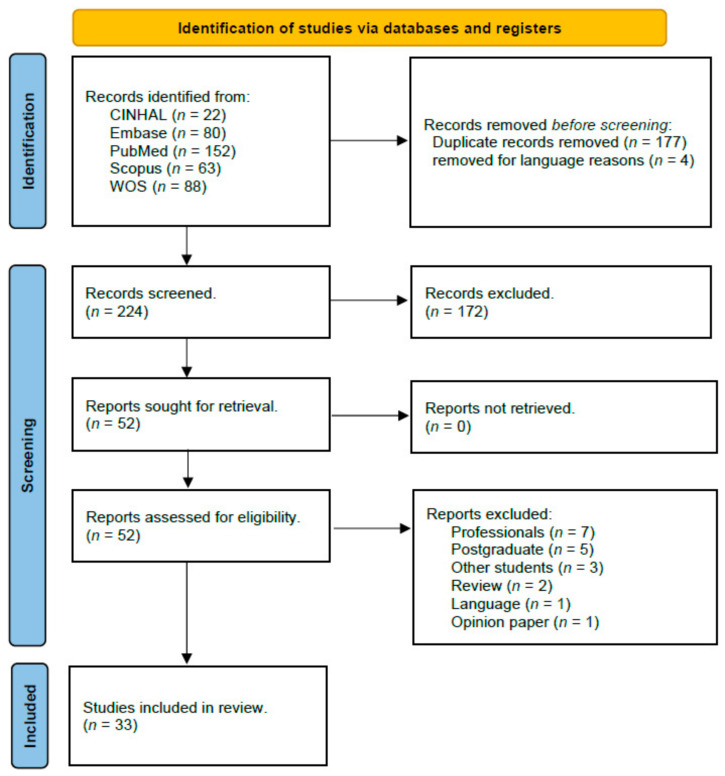
Prisma 2020 flow chart.

The initial search identified 405 potential articles. A total of 177 duplicate records were removed using EndNote software. Also, four articles were excluded for language reasons, as they were written in languages other than English or Spanish. After applying these filters and the predefined inclusion and exclusion criteria, 33 studies were included in the final review (Figure 2).

**Figure 3:**
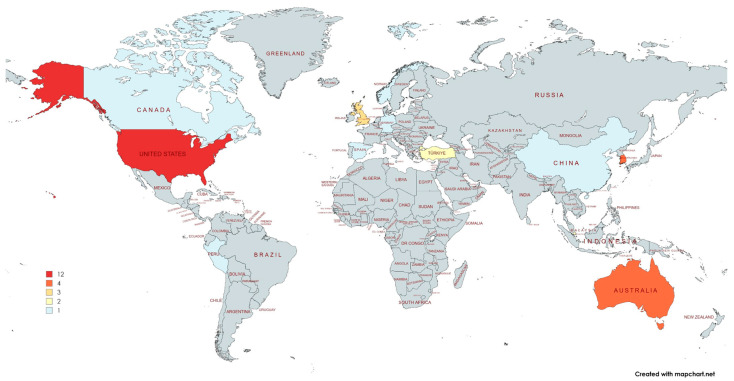
Distribution of papers on Mixed Reality in undergraduate nursing education by country.

These studies were distributed across various countries, with the United States (11 studies, 33%), South Korea (4, 12.1%), and Australia (4, 12.1%) being the most represented. Other contributing countries included the United Kingdom (3), Singapore (3), Turkey (2), and one study each from Canada, China, Germany, Norway, Peru, and Spain (Figure 3).

The number of publications on MR in nursing education has steadily increased, with an annual percentage change (APC) of 37.8% (Table S1). A notable shift occurred in 2020, where the previously rapid increase (APC = 56.7%) slowed to 13% per year (Figure S1).

### 3.2. Study Design

The included studies employed three primary research designs: quantitative (n = 17, 51.5%), mixed methods (n = 14, 42.4%), and qualitative (n = 2, 6.1%) (Table 1).

Among the quantitative studies, quasi-experimental designs were the most common (n = 20, 60.6%), followed by clinical trials (n = 9, 27.3%) and cross-sectional studies (n = 2, 6.1%). In the quasi-experimental category, pretest–posttest designs were the most frequently used: nine studies lacked a control group, while three included a control group (Table 1).

The sample sizes varied significantly across study designs, with an overall mean of 77.6 participants (SD = 79.2). Quantitative studies had a larger average sample size (98.4, SD = 100.99) compared to qualitative and mixed-methods studies (mean = 55.5, SD = 38.61), though this difference was not statistically significant (*p* = 0.179, Mann–Whitney U Test). Among quantitative designs, cross-sectional studies had the largest mean sample size (302 participants), followed by pretest–posttest studies with control groups (mean = 110 participants). Clinical trials, posttest-only designs, and pretest–posttest studies without control groups had smaller sample sizes (Table 2).

The studies were grouped according to the technological intervention applied to facilitate the interpretation of the findings. These categories include Immersive Virtual Reality (IVR) and Virtual Simulation (VS), Augmented Reality (AR), Mixed Reality (MR), mixed or blended simulations, Serious Games or Multi-User Virtual Environments (MUVEs), and other or conceptual interventions. The most common approach was IVR/VS, used in 11 out of 33 studies (33.3%), followed by AR in 9 studies (27.3%) and MR in 5 studies (15.2%). A summary of the distribution of studies by intervention type is presented in Table 3.

### 3.3. Evaluation of the Quality

#### 3.3.1. Quality Assessment of Pretest–Posttest and Cross-Sectional Studies Using the Newcastle–Ottawa Scale

In the quasi-experimental studies, the NOS scale for cohort studies was applied to 12 studies. In the selection criterion (maximum four stars possible), 10 studies scored two stars (✸✸) and two studies scored one star (✸). In the comparability criterion (maximum two stars), one study scored two stars (✸✸), and 11 studies scored one star (✸). In the outcome quality criterion (maximum three stars), three studies scored two stars (✸✸), six studies scored one star (✸), and three studies scored none. All longitudinal studies obtained at least a total score of three stars (Table 4).

In the cross-sectional studies, the adaptation of the NOS scale for cross-sectional studies was applied to 10 studies. In the selection criterion (maximum four stars possible), three studies scored one star (✸) and seven studies scored zero stars. In the comparability criterion (maximum two stars), eight studies scored one star (✸) and two had zero stars. In the outcome quality criterion (maximum three stars), one study scored three stars (✸✸✸), two studies scored two stars (✸✸), and seven studies scored one star (✸). All cross-sectional studies obtained at least a total score of three stars (✸✸✸) (Table 5). A minimum threshold of three stars on the Newcastle–Ottawa Scale indicated acceptable methodological quality, in line with commonly used criteria in systematic reviews.

#### 3.3.2. Risk of Bias in Clinical Trials Evaluated Using the RoB 2 Tool (Developed by the Cochrane Collaboration)

**Figure 4:**
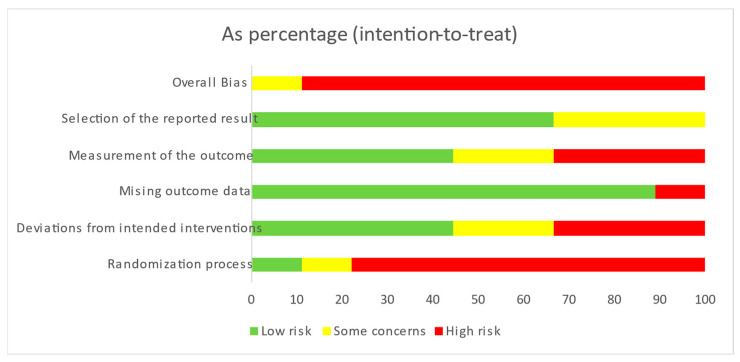
Risk of bias in clinical trials.

**Figure 5:**
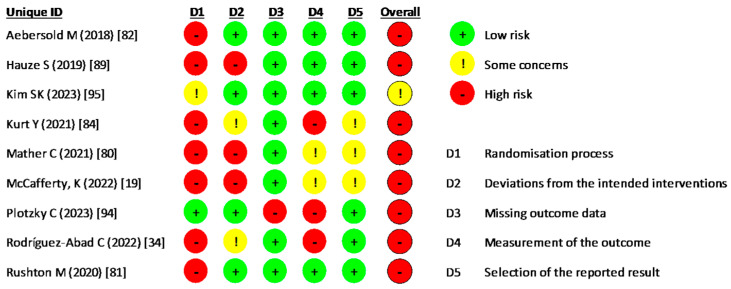
Risk of bias for each clinical trial included.

Based on the RoB 2 evaluation of the risk of bias in RCTs, 88.9% of the studies had a high risk of bias, 11.1% had a moderate risk, and none were categorized as having a low risk. Specifically, 44.4% of the studies exhibited a high risk of bias in the randomization process, while 33.3% demonstrated a high risk of bias about deviations from intended interventions and only 11.1% a high risk of missing outcome data. Regarding the measurement of outcomes, 66.6% had a low to moderate risk of bias in outcome selection, whereas 33.3% had a high risk (Figure 4). The detailed assessment for each study is illustrated in Figure 5.

#### 3.3.3. Quality Assessment of Qualitative and Mixed-Design Studies

Most of the 16 qualitative or mixed-design studies assessed using the JBICACQR demonstrated strong methodological quality across key domains (Table 6). A majority (62.5%) scored positively on philosophical congruity, with 31.3% marked as unclear, indicating room for more explicit alignment between methodology and philosophical underpinnings. All studies achieved the maximum score in objective congruity, method congruity, analysis congruity, and interpretation congruity, showcasing consistency and robustness. Regarding the researcher’s position and influence, the findings were mixed. Only 12.5% of studies explicitly addressed the researcher’s position, while the researcher’s influence was expressly considered by only 6.3% of studies. Regarding global ratings, 75% of studies were classified as high quality, while 25% were moderate, confirming the overall strength of the reviewed qualitative research.

### 3.4. Participants and Nursing Specialties

Most studies (57.6%) focused on students in a single academic year, while 21.2% included students from multiple academic years. Notably, 30.3% of the studies involved students in their second, third, and fourth years (Table 1). Additionally, 18.2% of the studies did not specify the academic year or semester in which the students were enrolled.

In 16 studies (48.5%), the students had clinical experience [22,75,76,77,78,81,83,84,86,91,92,93,95,96,97,100], although in one case, it was specified that they had no experience in Children’s Nursing. Four studies were conducted in mixed groups that included students with or without clinical experience [74,89,90] In the remaining studies, students either lacked clinical experience or had no clinical experience explicitly reported. Four studies were multicenter studies, conducted with students from several centers [19,80,83,89]. In some cases, they were students from several campuses of the same university, while in one case, they were students from several universities [83]. The others were conducted at a single university.

Study sizes had a median of 48 participants (quartile deviation = 33.5), varying from 5 to 414 participants (Table 1).

The articles covered a wide range of nursing specialties, including Critical Care Nursing [72], Medical–Surgical Nursing [82,97], Maternal–Child Nursing [73,83], General Nursing [88,89], Pharmacology [75], Community Health Nursing [76], Advanced Cardiac Skills, Arrhythmia and Electrocardiography [95], Clinical Nursing [8], Nurse Anesthesia [86], and Neurological or Critical Care Nursing [87].

Researchers focused some studies on nursing education rather than on any specialty [19,22,34,74,77,78,79,80,81,84,85,89,92,94,96,98,99] and Educational Technology [84,89]. Additionally, they examined Virtual Simulation in nursing education during COVID-19 [74,99].

### 3.5. Purpose of Using Mixed Reality

MR was predominantly employed to heighten the realism and authenticity of simulation-based education, bridging the gap between classroom learning and real-world clinical practice [8,19,72,80,83,84,87,88,95,97]. The superposition of virtual elements onto mannequins or the creation of fully immersive environments with avatars enabled students to visualize abnormal physiological cues and rehearse skills under conditions closely mirroring real-life scenarios. MR was used in mental health [93] and emergency care simulations [86], where interactive elements and dynamic patient responses deepened learners’ sense of presence and engagement [79,96].

Another objective was to foster essential nursing competencies such as clinical judgment, decision-making, and technical proficiency. Through repeated practice in a safe setting—such as Basic Life Support [81,88] or specialized procedures like leg ulcer management [19]—patient safety is ensured while students gain practical experience. Many studies further highlighted how MR-based simulations boost metacognition [98], improve academic performance [75,99], and enhance critical thinking [90,91,92,94]. In addition, they promote interprofessional communication [79] and collaborative problem-solving [22,74], thereby enriching the overall educational experience.

Several papers focused on MR’s role in reducing anxiety and strengthening students’ confidence as they transition into clinical roles [72,76,77,82]. Immersive simulations helped learners feel more prepared for challenging contexts, such as psychiatric care [72,93] or COVID-19-related patient management [92]. These findings align with literature on immersive disaster training, where students reported increased self-efficacy, creativity, and autonomy in decision-making without requiring constant instructor supervision [10]. Furthermore, recent research has shown that Augmented Reality can enhance simulation engagement and effectiveness, even when students participate as observers, highlighting the value of immersive tools in promoting active learning across different roles [101]. While increasing motivation and engagement [94], acceptance of these technologies appears high [85], with students viewing MR as a valuable tool for self-directed learning and clinical readiness [8,73,78].

### 3.6. Technology Used

VR was frequently employed to replicate clinical scenarios that might be difficult or unsafe to reproduce in real practice. For instance, researchers used IVR simulations to teach sepsis management [72] and pediatric clinical skills [73], while others used VR to explore mental health scenarios [93] and social determinants of health [76]. AR also featured prominently, with interactive modules for nasogastric tube placement [82], AR 360 photo-spheres for orientation [83], and advanced AR headsets like Microsoft HoloLens [89,97], merging virtual content with the real environment and supporting inter-professional learning [91], stroke care [97], and emergent procedures [86].

In these studies, technologies can be broadly categorized into wearable and non-wearable systems. High-fidelity simulations—using advanced mannequins or specialized software with avatars—further enriched hands-on practice. The subsections below delve into each of these categories in more detail.

#### 3.6.1. Wearable Technologies

Wearable devices—including Google Glass^®^, Oculus Go^®^, Meta Quest 2, and Microsoft HoloLens—allow users to experience realistic simulations and dynamic overlays. Two studies illustrated the use of wearable devices. In one study, students wore Google Glass^®^ to watch a pre-recorded scenario featuring a simulated patient while interacting with a mannequin, helping them contextualize the situation and focus on key clinical questions [102]. Another study employed Oculus Go^®^ headsets to present a series of 360° videos portraying actors with symptoms of schizophrenia in hospital or day-hospital rooms, allowing students to practice communication and recognize mental health indicators [93].

Several VR-based interventions incorporated Immersive Virtual Reality (IVR) to improve clinical competence. Studies [72,77,78,99] introduced simulations for procedures like intravenous therapy, sepsis management, and pediatric care using devices such as Meta Quest 2 and custom-built environments (Unreal Engine, Unity3D). VR tools also fostered cultural competence (e.g., interactions with a Bangladeshi patient [76]) and mental health training [93].

AR applications using wearable devices further contributed to skill development. For instance, AR overlays on mannequins displayed symptoms like facial drooping [87], while Google Glass^®^ enhanced the realism of respiratory deterioration scenarios [8].

#### 3.6.2. Non-Wearable Technologies

Non-wearable options encompass specialized rooms, 360° photo-spheres, and CAVE (Cave Automatic Virtual Environment) systems. Beyond VR, AR, and MR, 3D visualization systems, Serious Games, and Multi-User Virtual Environments (MUVEs) fostered active learning, critical thinking, communication, and teamwork. Non-wearable systems include immersive simulation rooms with realistic video projections and tracking systems. One study compared essential life support (BLS) training in an immersive simulation room—with stereoscopic images projected on walls and floors—to a CAVE system [102]. In the CAVE environment, users wore stereoscopic glasses, and head positions were tracked as they moved around the virtual scenario.

Another study developed a 360° AR photo-sphere to replicate a maternal–child clinical area, accessible via computers, smartphones, or tablets; learners could virtually manipulate objects such as cabinets and drawers 1 [83]. Additionally, an octagonal room called “Octave” provided the sensation of being on a busy street with traffic, allowing realistic interaction with virtual objects [81].

#### 3.6.3. Augmented Reality (AR) and Mixed Reality (MR)

Beyond wearable AR devices, smartphone-based AR apps reinforced anatomy, clinical procedures, and scenario-based learning [82,83,84]. For instance, AR posters used videos, images, and diagnostic reports [19] to bring clinical case scenarios to life, promoting active learning in areas like wound care [34].

MR using Microsoft HoloLens combines digital elements with the physical environment, enabling highly immersive simulations. Research by Frost et al. [90,91] and others [92] introduced HoloPatient in COVID-19 or stroke and tracheostomy scenarios [95,97], where students practiced clinical judgment and prioritization in hybrid real–virtual settings.

#### 3.6.4. Three-Dimensional Visualization Systems

High-end 3D visualization platforms also appear in nursing education. One study [75] compared a CAVE immersive 3D environment system to traditional 2D presentations for teaching pharmacological concepts, including drug-receptor binding. Another projection-based AR system [87] displayed dynamic facial expressions to mimic real-time symptoms, enhancing the realism of clinical simulations.

#### 3.6.5. Serious Games and MUVEs

The games provided structured scenarios that encouraged decision-making and clinical reasoning. One study [72] studied sepsis management using a game-based simulation. In another study, students practiced communication skills in Second Life, a multiplayer virtual world [98], which allowed them to practice in a safe virtual environment. These interactive platforms aimed to develop both technical and metacognitive skills.

#### 3.6.6. High-Fidelity Simulations

High-fidelity mannequins equipped with advanced software (e.g., QCPR for CPR training) offered realistic feedback on compression and ventilation performance [80,81]. Specialized MR systems, like Magic Leap One [86] and the Octave platform [81], simulated rare but critical emergencies—such as fires during clinical procedures—to prepare students for high-pressure, low-frequency events.

### 3.7. Teaching Results

The integration of MR in undergraduate nursing education has generated substantial interest due to its potential to enhance learning outcomes. Various studies have evaluated the effectiveness of MR-based teaching methods, focusing on key aspects such as knowledge acquisition, skill development, student engagement, and overall satisfaction. While many findings support the positive impact of MR on nursing education, some results remain inconsistent, highlighting its benefits and limitations. This section examines the primary outcomes observed in MR-based learning, including improvements in theoretical and practical competencies, its influence on student confidence and motivation, and the challenges associated with its implementation.

Table 7 summarizes the primary outcomes of meta-analyses conducted on MR-based teaching interventions in nursing education. It includes controlled and uncontrolled studies and reports the meta-analyses of several outcomes: knowledge, skills, anxiety, and trust. For each outcome, the table specifies the number of studies included (N), the model used (random effects or fixed effects), the overall effect size, the 95% confidence interval (CI), heterogeneity (I^2^), and Egger’s regression intercept with its CI to assess publication bias.

The meta-analyses of MR-based nursing education outcomes reveal varied effect sizes according to Cohen’s d criteria [103,104,105]. In controlled studies, knowledge showed a small effect (d = 0.093), skills a medium negative effect (d = −0.50), and anxiety a medium-to-large reduction (d = −0.73). Despite high heterogeneity, trust exhibited a large positive effect (d = 0.93). Uncontrolled studies demonstrated a medium effect on knowledge (d = 0.65). Egger intercepts indicate no significant publication bias across the analyses.

#### 3.7.1. Knowledge and Skill Acquisition

The systematic review and meta-analysis findings reveal notable differences in how MR interventions are perceived across different study designs. While qualitative and quantitative studies without a control group often report substantial improvements in students’ skills, knowledge, and clinical competence, the meta-analysis of controlled quantitative studies does not support a significant overall effect.

Many reviewed studies reported improved students’ knowledge, skills, and clinical competence through immersive technologies. VR-based interventions often target complex or high-risk scenarios, such as sepsis [72], pediatric emergencies [73], social determinants of health [76], and mental health care [93].

AR implementations likewise boosted procedural and clinical competencies; for instance, in nasogastric tube placement [82], orientation to new environments [83], and advanced cardiac skills [100]. Some projects combined AR with other modalities, including holographic MR [89,97] and 3D immersion systems [75], finding significant gains in posttest knowledge [34,75] and psychomotor skills [84].

MUVE simulations [98] and mixed-simulation formats [95] also led to measurable increases in clinical judgment, decision-making, and nursing competence. Although a few studies did not detect statistically significant differences in specific outcomes [77,80,81,94], the vast majority demonstrated clear learning benefits compared to more traditional teaching methods.

**Figure 6:**
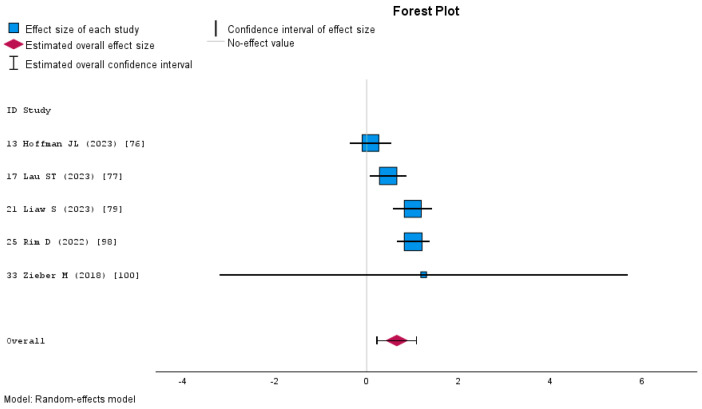
Forest plot of the meta-analysis of knowledge in studies without a control group.

MR shows a positive effect in uncontrolled studies. A random-effects model meta-analysis was conducted to evaluate the overall increase in knowledge after participating in a Mixed Reality learning activity in uncontrolled studies because the heterogeneity analysis revealed substantial variability among studies (I^2^ = 73%). The pooled effect size was moderate 0.656 (95% CI: 0.226–1.086, *p* = 0.003), indicating a moderate improvement in knowledge following the intervention (Figure 6).

**Figure 7:**
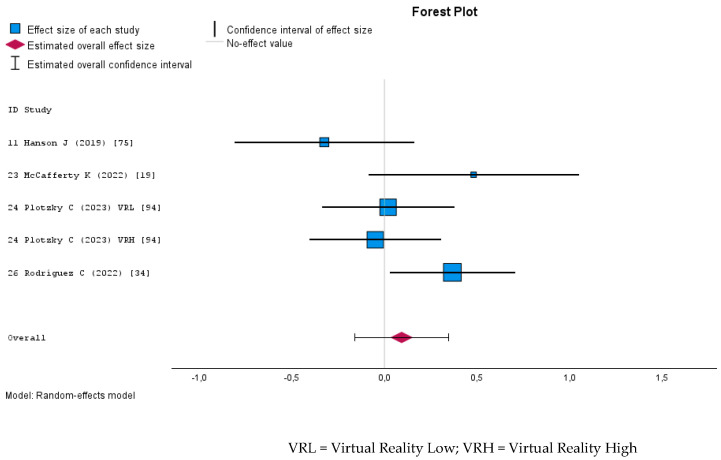
Forest plot of the meta-analysis of knowledge in controlled studies.

A meta-analysis assessing the impact of Mixed Reality on knowledge acquisition in nursing education was computed using a random-effects model due to the moderate heterogeneity observed among the included studies (I^2^ = 47%). The overall estimated effect size, measured by Cohen’s d, was 0.099 (95% CI: −0.168 to 0.367), which was not statistically significant (*p* = 0.466). According to conventional interpretations of Cohen’s d, this represents a negligible effect size [106], suggesting that Mixed Reality yielded no meaningful improvement in knowledge acquisition compared to traditional or alternative educational methods. Figure 7 presents the forest plot, displaying the individual effect sizes and confidence intervals.

**Figure 8:**
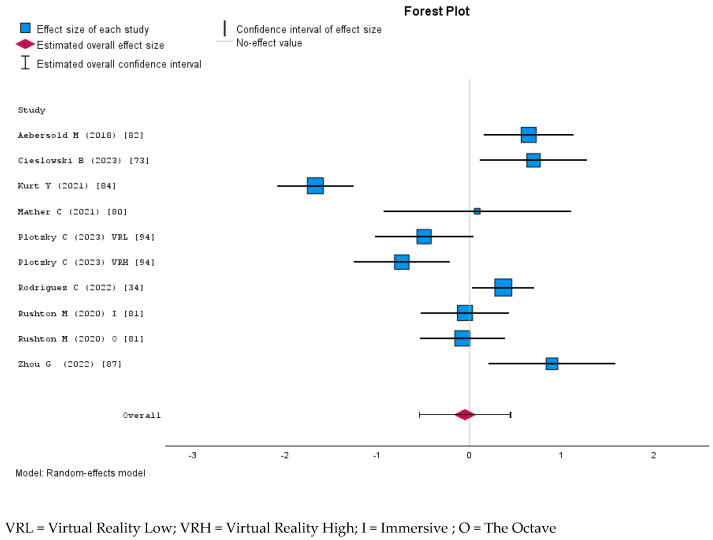
Forest plot of the meta-analysis of skills in controlled studies.

MR interventions did not significantly improve students’ skills compared to traditional or other digital learning methods. The random effect meta-analysis examining the effect of MR on nursing students’ skills yielded a negligible overall effect size of d = −0.05 (95% CI: −0.54 to 0.44), which was not statistically significant (*p* = 0.84). The heterogeneity was high (I^2^ = 89% Q = 94.78, *p* < 0.001), indicating substantial study variability. The Egger’s test for publication bias was not significant (*p* = 0.46), suggesting no firm evidence of small-study effects. The forest plot (Figure 8) illustrates the individual study effect sizes, showing a wide range of estimates, with some favoring Mixed Reality and others favoring the control conditions.

While some studies showed a slight positive trend, the high variability indicates the need for additional research with larger samples and robust methodological designs to draw definitive conclusions. The low tau-squared estimate (τ^2^ = 0.04) suggests that the between-study variance is not excessive, although some heterogeneity justifies using the random-effects model.

#### 3.7.2. Self-Confidence, Anxiety, and Motivation

Many papers highlighted increased self-confidence, anxiety reduction, and heightened motivation associated with immersive teaching [72]. Four studies measured anxiety [22,72], although only two studies were with a control group [55]. In all cases, students in the MR group exhibited reduced anxiety levels [95].

**Figure 9:**
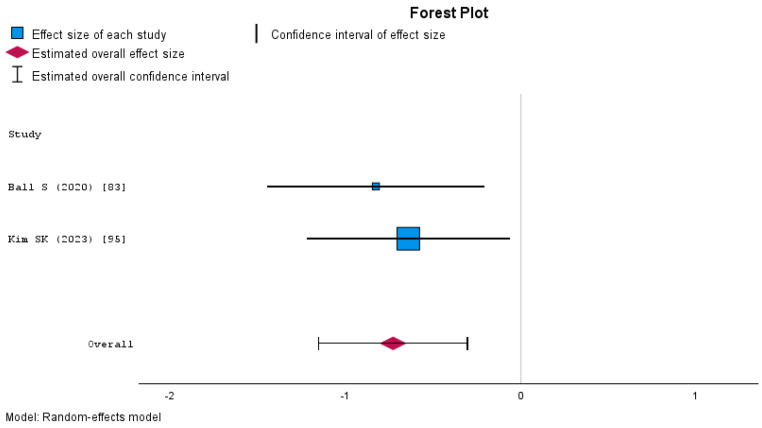
Forest plot of the meta-analysis of anxiety.

The fixed-effects meta-analysis on anxiety yielded an overall effect size of Cohen’s d = −0.73 (95% CI: −1.15 to −0.31, *p* < 0.001), indicating a significant reduction in anxiety following the intervention (Figure 9). Heterogeneity was minimal (I^2^ = 0.00%), suggesting consistency across the included studies.

**Figure 10:**
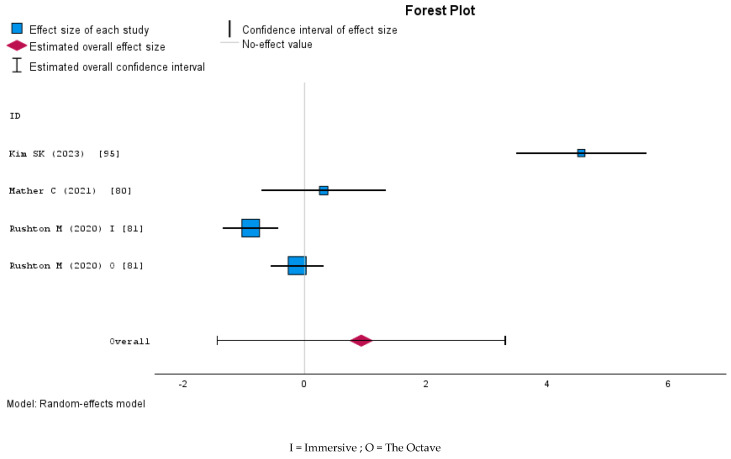
Forest plot of the meta-analysis of trust.

A random-effects meta-analysis was conducted on four studies, each assessing multiple dimensions of trust. Pooled estimates of the mean and standard deviation were calculated for these dimensions, yielding an overall effect size (Cohen’s d) of 0.93 (SE = 1.21, 95% CI [−1.44, 3.30], *p* = 0.44). The results indicate that, although the combined estimate suggests a positive effect, it was not statistically significant (Figure 10). Moreover, heterogeneity was substantial (tau^2^ = 5.67, I^2^ = 0.98), reflecting considerable variation among the individual studies’ measurements or populations.

Nevertheless, students reported feeling more confident in interprofessional communication after using an AI-enabled VR simulation [79].

Students emphasized the motivational value of MR activities [89,90] or the way VR simulations helped them feel genuinely immersed in clinical scenarios [92,93]. In some cases, AR-based tools provided consistency or standardized experiences, such as orientation in a 360-degree photo-sphere [83], reducing stress in new clinical settings.

Studies examining decision-making [95] and advanced clinical skills [96] reported positive learner confidence and competence shifts. Even in environments perceived as initially intimidating [81,86], the immersive nature of the simulations prompted students to adapt and sharpen emotional resilience.

#### 3.7.3. Student Satisfaction and Technology Acceptance

The relationship between satisfaction and academic performance was not always direct [99], suggesting that high enthusiasm for virtual learning may not automatically translate into higher grades.

Nonetheless, satisfaction scores for AR posters [19], low-cost AR stroke simulations [100], and MR simulation programs [98] were consistently favorable, reflecting an appreciation for safe, innovative, and realistic learning environments.

Learner satisfaction was broadly high across technologies, yet some nuances emerged. Technology acceptance also remained robust, even in scenarios where video-based methods occasionally outperformed VR for psychomotor skills [94] or where a CAVE environment introduced distractions [81].

### 3.8. Challenges and Limitations of Mixed Reality in Nursing Education

While Mixed Reality (MR) offers significant educational benefits, its implementation is not without challenges. These can be broadly categorized into adverse physiological effects experienced by students and technological and usability challenges that affect the learning process.

#### 3.8.1. Adverse Physiological Effects

Challenges such as device unfamiliarity [69,78], AI responsiveness [72], and comfort with head-mounted displays [70] repeatedly surfaced, underscoring the importance of comprehensive training and support.

While students routinely endorsed the realism and interactivity of vSim [22] HoloLens [89] and hybrid AR/VR platforms [8,95], specific cohorts experienced motion sickness or physical discomfort [77,78,93].

Several studies reported cybersickness symptoms, including dizziness, nausea, and visual discomfort when using MR devices. Participants in immersive VR simulations experienced neck strain and disorientation, often linked to the weight of headsets and prolonged exposure [72,77,78,81,90,93,94,97,100]. When VR glasses were used for extended periods, motion sickness was reported frequently. Studies recommend limiting head-mounted displays to 15–20 min to reduce discomfort [93]. Some students struggled to focus, particularly in AR simulations [8].

#### 3.8.2. Technological and Usability Challenges

Beyond physical discomfort, technical difficulties presented significant barriers to MR adoption in nursing education. Slow system performance, overheating, and connectivity issues were frequently cited as problems that hindered smooth learning experiences [8,74,92]. Some students struggled to navigate virtual environments due to unresponsive controls, low-resolution graphics, or poor audio synchronization [22,93]. In Augmented Reality-based simulations, errors in server connections, missing content, and difficulty locating necessary features further complicate the experience [97].

The success of “helping hands” tele-assistance [88] and advanced projection-based systems [87] highlights the value of real-time guidance and feedback in mitigating these issues. Additionally, hardware limitations posed a challenge. Some MR headsets blocked peripheral vision, while tactile feedback—a critical clinical training component—was missing in many simulations [97].

#### 3.8.3. Pedagogical and Instructional Considerations

Studies covering emergent procedures [81] and COVID-19 patient care [79] illustrate the ongoing expansion of VR and AR into high-acuity, low-frequency situations, reinforcing the potential for immersive simulations to fill critical gaps in clinical exposure. Several studies highlighted the importance of user training to maximize MR effectiveness. In an AR-based nasogastric tube placement module, participants criticized the redundancy of the content and noted that access to devices was limited, restricting their ability to practice independently [82]. Some MR simulations required explicit guidance on how to interact with virtual environments, particularly in tele-assistance models where students depended on near-eye displays for remote coaching [88].

While MR enhances realism, some students perceive a gap between expected and actual learning outcomes. AI-driven simulations, for example, were criticized for their lack of human-like interaction in virtual patient encounters [79]. Furthermore, logistical challenges—such as coordinating faculty training, scenario design, and integration into existing curricula—were cited as ongoing obstacles [96].

#### 3.8.4. Cost and Accessibility Issues

The high cost of MR technology may limit its widespread adoption in nursing education [92]. MR-based training setups, including head-mounted displays, AR-equipped mannequins, and high-fidelity simulations, were often cited as financially demanding, making them less accessible to institutions with limited budgets.

Some studies noted differences in cost-effectiveness between MR-based approaches and traditional simulation methods. For example, an MR-based tracheostomy fire simulation costs USD 12,500, while a traditional high-fidelity simulation using theatrical fog was estimated at only USD 75 [92]. These costs stem from hardware (e.g., headsets) and specialized software development, including programming, content creation, and maintenance. These disparities highlight the need for cost-effective, scalable solutions tailored to different educational settings.

The availability of MR devices also varied across studies, with some reporting limited access due to shared equipment, lack of dedicated MR spaces, and insufficient faculty training [78,81]. In some cases, students reported restricted opportunities to use MR tools due to logistical constraints [54].

A few studies mentioned challenges in integrating MR into the curriculum, particularly in institutions with limited technological infrastructure or lack of faculty expertise in MR-based teaching methodologies [96].

## 4. Discussion

Many papers originate from English-speaking high-income countries —namely, the United States, Australia, and the United Kingdom—contributing over half of the included studies (55.8%). This pattern aligns with findings from previous reviews in other healthcare contexts, such as RA in ICUs [11].

A key research question concerns the potential long-term implications of MR for undergraduate nursing education. Although short-term findings from multiple studies indicate that MR-based strategies can reduce learner anxiety and enhance clinical competence, less is known about the durability and scalability of these benefits. Whether students trained with MR achieve superior long-term performance or find it easier to adapt to the clinical workplace upon graduation remains unclear. MR could influence the structure of nursing curricula, creating immersive, highly interactive environments that will allow students to engage in repeated practice of complex procedures, experience virtual simulations of rare clinical scenarios, and develop interprofessional communication skills in a controlled yet authentic context. There is also a growing need to standardize MR implementation across educational settings to ensure consistent learning experiences. Future longitudinal research and large-scale randomized controlled trials will be essential to ascertain how sustained use of MR might reshape nursing competencies, foster more robust critical thinking, and ultimately improve the quality of patient care [107]. Most of the educational implementations described are focused on training professionals—particularly in essential life support [80,108,109]—and in basic sciences such as anatomy [110,111,112] and pharmacology [75]. The diverse range of specialties in which MR was used reflects the growing interest in using VR and AR across various nursing fields.

### 4.1. Limitations

This review has several limitations. The sample sizes of the included studies were generally small and highly variable, limiting the generalizability of the findings. Second, the heterogeneity in study design and outcome measures hindered intervention standardization, complicating comparisons and synthesis. These limitations underscore the need for future research with larger, more standardized, and methodologically rigorous designs to understand Mixed Reality’s role in nursing education. Despite the increasing interest in simulators and MR, few thorough evaluations exist, and many studies remain pilot projects with minimal sample sizes or even single-participant designs [80,113,114,115,116,117].

A major challenge encountered in this review is the lack of standardization and clarity surrounding the concepts and definitions of AR, VR, and MR [118] across studies, making it challenging to evaluate the specific impact of each technology. One conceptualization places these technologies on a continuum from real to virtual environments, with Augmented Reality defined as a technology that supplements real-world views with simulated cues. In its narrowest sense, Augmented Reality uses transparent screens or glasses to enable a direct view of the physical environment [119]. The confusion partly arises because certain companies market their products as “mixed reality” or “augmented reality” devices for promotional reasons, regardless of the underlying technology [17].

#### 4.1.1. Limitations of Technology

Despite the promising advantages of Mixed Reality (MR) for nursing education, several technological and human–technology interaction limitations warrant further investigation. One crucial concern involves the physical discomfort some learners experience, including eyestrain, dizziness, and general fatigue—symptoms collectively referred to as “cybersickness”—which are common in immersive VR settings [30]. MR devices have been associated with visual discomfort, dizziness, and headaches in students and nurses, suggesting the need for controlled usage durations [114,120]. These issues can negatively impact students’ immersion and overall learning outcomes. Future studies should, therefore, explore strategies to mitigate discomfort, such as optimizing headset ergonomics, adjusting visual parameters (e.g., brightness and field of view), and incorporating breaks or guided rest periods during simulations.

Additionally, the complex interplay between users and technology remains insufficiently understood, particularly when novices are exposed to advanced MR devices. Variations in motor skills, perceptual processing, and user confidence can all influence training efficacy [121]. Integrating human factors and engineering principles into MR design could address these challenges by systematically evaluating ergonomic, cognitive, and environmental factors. Such an approach would help ensure that technology serves curricular objectives and is usable, accessible, and tailored to diverse nursing student populations. By emphasizing both physical comfort and improved human–tech interaction in future research, educators and developers can further refine MR systems to maximize their educational impact.

Another issue is the selection of participants in some studies. Although in some studies, the original aim was often to reduce the anxiety of novice students entering internships, several interventions were carried out on students who already had clinical experience and had completed some or all their internships. This is a threat to external validity, as these experienced students differ from the intended population—those with no clinical exposure who would stand to benefit most from MR-based simulations. Future research should target students without clinical experience, using well-designed controlled trials with adequate sample sizes and validated metrics of teaching effectiveness. Examples of such instruments include the Instructional Materials Motivation Survey (IMMS), validated for MR [122], and tools for evaluating psychomotor skill acquisition [123].

Experience in other health professions can also inform nursing education research. MR has been used in medical education to foster empathy, allowing students to experience terminal illness diagnoses and observe family reactions [124] and to facilitate interprofessional collaboration [108,115,117,125,126]. It has even been proposed that MR could help students train for culturally diverse contexts in global and international nursing [127]. One limitation of our review is the absence of studies assessing the long-term outcomes of immersive technology-based nursing education [107]. Other emerging topics include using VR and virtual patients for communication skills training [128,129].

Many uncontrolled studies highlight the advantages of immersive technologies in complex and high-risk scenarios, showing promising results in knowledge acquisition and psychomotor performance. In contrast, in the meta-analysis, the pooled effect size from controlled trials suggests that MR interventions do not outperform traditional or other digital learning methods. This discrepancy may stem from differences in study methodologies, outcome measures, and the potential influence of publication bias or positive reporting tendencies in uncontrolled research.

#### 4.1.2. Publication Bias and Methodological Challenges

We did not detect publication bias. Publication bias may also skew the current findings, as studies reporting the significant positive effects of Mixed Reality (MR) are more likely to be published. Future research could additionally employ Trim-and-Fill methods to further evaluate and adjust for publication bias, thereby refining the accuracy of meta-analytic estimates. Methodological heterogeneity, including varied sample sizes, intervention protocol inconsistencies, and outcome measure differences, posed a significant challenge, limiting comparability and synthesis across studies. Standardizing protocols and metrics across studies will strengthen evidence of MR’s effectiveness in nursing education.

#### 4.1.3. Comparison Between Qualitative and Quantitative Results

In qualitative research, findings are typically expressed in terms of emergent themes or categories rather than statistical metrics, and sampling continues until data saturation is achieved when no new information emerges [130]. Quantitative research aims for statistical significance by measuring outcomes within a pre-established sample size, which can reveal whether observed effects are statistically significant. This fundamental methodological divergence helps clarify why, in this study, qualitative studies of MR consistently showed substantial thematic benefits. Students often reported deeper engagement, immersion, reduced anxiety, and learning, yet these outcomes may not always translate into significant quantitative findings. Moreover, heterogeneity in MR technologies (e.g., varying headset software platforms) and small sample sizes, coupled with inconsistent evaluation methods, can diminish statistical power and mask potential improvements in measured outcomes [131,132]. A more robust approach, incorporating standardized measures, larger multi-site samples, and more precise definitions of MR interventions, could better unpack the discrepancy between the consistently positive qualitative experiences and the uneven quantitative evidence, strengthening the overall discussion.

### 4.2. Integration of AR and MR in Nursing Education and Future Prospects

In nursing-specific scenarios, AR and MR have been employed to assist operating room nurses in orthopedic surgery [116], neurosurgery [120,133], ophthalmic surgery [134], Basic Life Support (BLS) [80,108], pediatric patient transport [117], pain unit management [105], and ultrasound scanning [135]. VR has prepared students to care for patients with infectious diseases [136] and pressure injury assessment [137]. Since MR provides instant feedback, it can potentially enhance the learning process [8]. A meta-analysis of 23 studies [107] found that immersive technologies can significantly improve nursing students’ knowledge, self-efficacy, and confidence, aligning these findings with the New World Kirkpatrick Model framework [138]. VR can emotionally impact [139], establishing connections and empathy. VR has been used in mental health, providing students with the emotional feeling of affection and closeness [140]. In Lee’s study, the students were in a setting with a patient with schizophrenia using a head-mounted display. The simulation included scenarios of risk of auditory hallucination, violation, visual hallucination, delusion, and risk of suicide. This allowed the student to interact in a dangerous situation without risk and better understand schizophrenia [87]. Students preferred MR-based training on emergencies, urgent care, medication management, and respiratory procedures [22]. Before fully integrating these techniques into curricula, it is crucial to develop robust methods for assessing competencies and outcomes, as traditional assessments may be insufficient [118].

MR may eventually find therapeutic applications in nursing care, but this is out of the scope of this review. For instance, using MR in nursing homes has been proposed to improve resident engagement [141]. Thus, incorporating MR into nursing education could equip future nurses with valuable, forward-looking skill sets.

The absence of differences in the knowledge detected in our meta-analysis of controlled studies is consistent with the results of other studies on MR [137,142]. On the other hand, a meta-analysis of VR in nursing education that has knowledge as a secondary outcome found improvements [143]. The positive effects of Mixed Reality (MR) reported in the literature and other systematic reviews may overestimate its impact in uncontrolled studies [144]. In our analysis, MR appeared to enhance knowledge acquisition in pretest–posttest studies without a control group, but when compared to traditional or alternative digital learning methods in controlled studies, no significant differences were observed. The learning gains attributed to MR may partially reflect a general effect of structured educational exposure rather than a specific advantage of the technology. This emphasizes the importance of well-designed controlled studies evaluating MR’s impact on learning outcomes.

Cost issues make it challenging to deploy VR, especially in developing countries. There are substantial hardware acquisition, software development, and implementation costs. The number of VR headsets sold yearly worldwide is around 10 million. For instance, MR simulations may range from USD 75 using theatrical fog to over USD 3500 with advanced headsets like the Apple Vision Pro or Microsoft HoloLens [92,137,145,146].

Finally, it is essential to remember that MR is not a universal solution for educational challenges in health sciences [123,147]. A recent mixed-methods evaluation demonstrated that while virtual simulations can effectively augment learning (particularly during disruptions like the COVID-19 pandemic [148,149], user satisfaction and implementation efficacy depend on multiple factors [150]. Similarly, another research study showed that Augmented Reality can bolster student engagement and learning in complex cardiac topics [151]. As with any educational tool, strategic and context-specific decisions must guide MR implementation to ensure it meaningfully enhances nursing education [86].

### 4.3. Ethical Considerations in the Use of Mixed Reality in Nursing Education

One aspect that should be considered in the future is the ethical aspects [152]. When MR is incorporated into regular teaching, students should be informed of potential problems, such as cybersickness. Subsequently, students should be asked for their consent. Students should have the right not to participate for health reasons (e.g., epilepsy, motion sickness) or simply because of beliefs. Participation in MR should be voluntary, and students should have an alternative to this practice [153].

In the future, it will be necessary to study whether prolonged exposure to MR can create psychological problems such as stress or cognitive overload [154], especially in simulations related to the ICU or CPR [155]. On the other hand, exposure to virtual patients and emergencies could cause some insensitivity to human pain, and MR training should also be used to promote empathy in students. Therefore, the professional ethical and deontological guidelines of nursing should be respected. Ethical issues should also be included in simulation scenarios.

### 4.4. Recommendations for Future Research

Future studies should prioritize randomized controlled trials with adequate sample sizes and standardized outcome measures. Moreover, researchers should work toward a unified theoretical framework that clearly distinguishes between MR, AR, and VR technologies and defines their pedagogical roles [156]. Such a framework will help enhance comparability across studies and support evidence-based integration of immersive technologies into nursing curricula. Additionally, face-to-face simulation remains crucial to ensure real-world interpersonal skills and teamwork abilities are effectively developed alongside emerging digital methods [157]. Finally, meta-analyses focusing on high-quality studies could provide more definitive conclusions regarding the relationship between Mixed Reality interventions and learning outcomes [158].

To fully harness the potential of MR in nursing education, future studies should explicitly align simulation designs with constructivist or experiential learning theories. For example, Kolb’s Experiential Learning Cycle underscores active, self-directed engagement, reflective observation, and conceptual application, aligning naturally with MR’s immersive capabilities [159,160,161]. Similarly, integrating Vygotskian principles—such as scaffolding and the zone of proximal development—can help tailor MR-based tasks to learners’ individual needs, ensuring they advance from foundational skills to complex clinical reasoning [162,163,164,165,166,167]. Grounding MR curricula in established learning theories, such as Kolb’s Experiential Learning Cycle and Vygotsky’s zone of proximal development, may enhance instructional effectiveness and foster deeper learning, critical reflection, and long-term skill retention, thereby strengthening the pedagogical foundations of MR-based nursing education [168,169,170].

It is necessary to demonstrate the effectiveness of MR in specific settings, such as CPR or emergency departments, with randomized controlled studies and with an adequate sample size.

A problem during this study was the lack of standardization in the information presented; only a few studies presented the mean and SD. The SD had to be deduced through mathematical formulas or by measuring graphs. The lack of standardized reporting and limited availability of raw data complicated our analysis, underscoring the importance of open data policies for future meta-analyses. In this sense, the extension of open data policies is important, as this will facilitate the performance of meta-analysis.

## 5. Conclusions

MR offers a safe, immersive environment where nursing students can practice clinical skills, enhance critical thinking, build confidence, and reduce anxiety in clinical settings. Despite its potential, MR in nursing education faces several limitations, including physical discomfort, usability challenges, and high implementation costs.

This systematic review used established quality assessment tools to investigate the relationship between the quality of the included studies and their intervention outcomes. A notable proportion of the reviewed studies showed a high risk of bias or concerns regarding methodological rigor. Nevertheless, most demonstrated positive learning benefits from MR interventions compared to traditional teaching methods. The absence of a clear association between study quality and intervention effect size could be attributed to the limited number of studies or the heterogeneity in intervention designs and outcome measures. It is conceivable that investigations with stronger methodological quality and reduced risk of bias might reveal even more substantial positive effects of MR interventions.

While qualitative studies consistently reported enhanced engagement and reduced anxiety, quantitative findings were often negligible or non-significant, likely due to small sample sizes and inconsistent protocols. Consequently, standardized and rigorously controlled trials must validate and quantify these perceived benefits.

Overall, MR shows considerable promise for enhancing engagement, reducing anxiety, and developing clinical competencies among undergraduate nursing students. However, its comparative effectiveness remains inconclusive, primarily due to technological heterogeneity, small sample sizes, and inconsistent outcome measurements. Future research employing standardized protocols and larger, more rigorously controlled trials is essential to determine whether the qualitatively observed benefits of MR can be robustly translated into significant and reproducible quantitative improvements in nursing education. Future research should also evaluate its long-term impact on nursing competence and explore its potential across diverse nursing specialties and educational contexts.

## Figures and Tables

**Table 1:** Summary of included studies: author, country, design, and participants, sorted by type of intervention.

Author (Year of Publication)	Country	Design	Qualitative	Quantitative	N VR	N Control	Courses
Immersive Virtual Reality (IVR)/Virtual Simulation (VS)							
Foronda CL (2018) [22].	USA	Mixed	Qualitative content analysis ^†^	Post (no CG)	99	No	-
Adhikari R (2021) [72]	UK	Mixed	Focus group	Pre–Post (no CG)	19	No	3
Cieslowski B (2023) [73]	USA	Quantitative		Post (no CG)	24	24	-
Flo J (2021) [74]	Norway	Mixed	Focus group	Pre–Post (no CG)	33	No	2
Hanson J (2019) [75]	Australia	Quantitative		Pre–Post (CG)	184	18	2
Hoffman JL (2023) [76]	USA	Mixed	Qualitative content analysis	Pre–Post (no CG)	100	No	-
Lau ST (2023) [77]	Singapore	Mixed	Focus group	Pre–Post (no CG)	34	No	1
Lau ST (2023) [78]	Singapore	Mixed	Qualitative content analysis	Post (no CG).	29	No	2
Liaw S (2023) [79]	Singapore	Mixed	Focus group	Pre–Post (no CG)	32	No	4
Mather C (2021) [80]	UK	Quantitative		Clinical Trial	7	8	1
Rushton M (2020) [81]	UK	Mixed	Qualitative content analysis	Clinical Trial	80, 73	55	53
Augmented Reality (AR)							
Vaughn J (2016) [8]	USA	Quantitative		Pre–Post (no CG)	12	24	1–2
Aebersold M (2018) [82]	USA	Quantitative		Clinical Trial	35	34	2–3
Ball S (2020) [83]	USA	Quantitative		Pre–Post (CG)	30	17	3–4
Kurt Y (2021) [84]	Turkey	Quantitative		Clinical Trial	64	58	1
McCafferty, K (2022) [19]	USA	Quantitative		Clinical Trial	15	64	1
Rodríguez-Abad C (2022) [34]	Spain	Quantitative		Clinical Trial	72	65	2
Uymaz P (2022) [85]	Turkey	Quantitative		Cross-sectional	419	No	-
Wunder L (2020) [86]	USA	Quantitative		Pre–Post (no CG)	33	No	
Zhou G (2022) [87]	USA	Mixed	Qualitative content analysis	Pre–Post (CG)	18	No	
Mixed Reality (MR)/Holographic Simulations							
Barnett T (2017) [88]	Australia	Quantitative		Post (no CG)	5	No	5
Hauze S (2019) [89]	USA	Quantitative		Clinical Trial	53	108	1, 2, 3, 4
Frost J (2020) [90]	Australia	Quantitative		Post (no CG)	96	No	2
Frost J (2020) [91]	Australia	Qualitative	Directed content analysis	-	6	No	-
Son Y (2023) [92]	Korea	Qualitative	Focus group		30	17	4
Mixed Simulations (360°/High-Fidelity/Blended)							
Lee Y (2020) [93]	Korea	Mixed	Thematic analysis	Post (no CG)	60	No	4
Plotzky C (2023) [94]	Germany	Mixed	Focus group	Clinical Trial	47	84	2–3
Kim SK (2023) [95]	Korea	Quantitative		Clinical Trial	25	23	
Lam VSF (2020) [96]	China	Quantitative		Post (no CG)	35	No	4
Liang C (2021) [97]	USA	Mixed	Content analysis	Post (no CG)	85	No	4
Serious Games/v							
Rim D (2022) [98]	Korea	Mixed	Focus group	Pre–Post (no CG)	57	No	4
Other/Conceptual/Not classified							
Curro-Urbano O (2022) [99]	Peru	Quantitative		Cross-sectional	186	No	2, 3, 4
Zieber M (2018) [100]	Canada	Mixed	Inductive thematic analysis	Pre–Post (no CG)	24	No	3–4

^†^ Sandelowski’s method; post (no CG) = posttest without a control group; pre–post (no CG): pretest–posttest design without a control group; pre–post (CG): pretest–posttest design with a control group; MUVEs = Multi-User Virtual Environments.

**Table 2:** Summary of included studies: author, outcome, magnitude, and significance, sorted by type of intervention.

Author (Year)	Intervention	Control	Context	Outcome Measurements	Effect Intervention vs. Control	*p*
Immersive Virtual Reality/Virtual Simulation						
Adhikari R (2021) [72]	IVR and gamification	No	Managing a case of pneumonia with sepsis	NASC-CDM	Self-confidence (26.1%) ^†^; anxiety (−23.4%)	*p* < 0.001 *p* < 0.001
Cieslowski B (2023) [73]	Immersive Virtual Reality (IVR) simulation training on acute pediatric care using six scenarios	Usual training	Pediatric clinical course	Performance outcomes measured (infection control, initial assessment, oxygen therapy, and total performance)	Moderate-to-large (d = 0.69) Infection control (62.5 vs. 47.9) Initial assessment (59.38 vs. 45.31) Oxygen therapy (32.29 vs. 12.50)	*p* < 0.05
Flo J (2021) [74]	VS using Body Interact™ through Zoom	No	Nursing students	Skill development Simulation engagement Learning confidence Diagnostic accuracy Team collaboration Reflection and debriefing	Self-confidence (5.6 vs. 5.1); Reasoning skills (5.3 vs. 4.6); Learning gap bridging (5.6 vs. 4.8) Decision-making (6.1 vs. 5.6)	*p* = 0.004 *p* = 0.001 *p* = 0.001 *p* = 0.016
Foronda CL (2018) [22]	vSim for Nursing™ virtual simulation for medical–surgical learning	No	Nursing education in a clinical setting	Knowledge improvement, confidence, self-reported perceptions of preparedness	Knowledge scores: 73.31 vs. 65.36; anxiety levels: 73.26 vs. 57.75	*p* = 0.032 *p* = 0.002
Hanson J (2019) [75]	3D immersive visualization technology (using CAVE2™)	2D wide-screen visualization in a standard teaching space	Pharmacodynamic concepts, drug-receptor binding	Pre- and post-intervention knowledge tests Student discomfort Student satisfaction	Knowledge acquisition improvement 3D = 1.16; 2D = 0.55 Proportion of correct answers 3D = 25.3%; 2D = −9.1%	*p* = 0.001 *p* = 0.008
Hoffman JL (2023) [76]	SimX VR medical simulation with Oculus Quest 2 business VR headset and hand controllers.	No	A simulated scenario of a community health nurse conducting a clinic for a Bangladeshi man at a local mosque	Knowledge SDOH Cultural competence Students’ perceived learning and confidence Effectiveness simulation phases	Pretest: mean = 10.8, SD = 5.28; Posttest: mean = 11.06, SD = 4.97	*p* = 0.720
Lau ST (2023) [77]	IVR Design and evaluation with respect to using head-mounted VR for learning clinical procedures	No	IV therapy, SC insulin injection	Knowledge improvement of clinical procedures Game perception User reaction	Overall, knowledge (not available) SC insulin knowledge (not available)	*p* = 0.075 *p* = 0.042
Lau ST (2023) [78]	IVR Using IVR for clinical skills learning for mid-career switch students	No	IV therapy, SC insulin injection	Usability Scale (SUS) Perception of continuance intention Qualitative feedback on user experience and challenges	Not applicable	NA
Liaw S (2023) [79]	AI-enabled VRS	No	clinical deterioration of patients in a virtual hospital ward, interprofessional communication	Communication Knowledge, PIE-SES, TAM, and API Focus group discussion	Communication knowledge, 32.46%; interprofessional communication self-efficacy, 13.71%	*p* < 0.001 *p* < 0.001
Mather C (2021) [80]	IVR in ER with sound props: mannequin dressed in a hospital gown, hospital bed, cardiac arrest trolley, and clinical equipment	Standard classroom ’Little Anne’ mannequin (not dressed)	CPR	Confidence Performance	Confidence training in CPR = 31.20% Overall quality of CPR −5%	*p* = 0.025 *p* = 0.711
Rushton M (2020) [81]	IVR low-simulation room with video IVR high-fidelity immersive system “The Octave”	Non-immersive	Basic Life Support (BLS) in emergencies	Confidence levels Competence and skills scores	Gain confidence initiating CPR Ctrl/IVR low: −60%; high: −20% Gain confidence in mask ventilation Ctrl/IVR low: −49.32%; high: −48.63% Compression hand positioning Ctrl/IVR low: −9.73%; high: −21.41%	*p* = 0.001 *p* < 0.001 *p* = 0.001
Augmented Reality (AR)						
Vaughn J (2016) [8]	Google Glass (ARH) to project videos into the student’s field of vision during a high-fidelity simulation	No	Asthma exacerbation and respiratory distress in a mannequin-enhanced environment	simulation design Self-confidence in learning	Not applicable	NA
Aebersold M (2018) [82]	iPad Anatomy-Augmented VS	Usual training	Placement of a nasogastric tube (NGT)	Competency in NGT Placement	3.70%	*p* = 0.011
Ball S (2020) [83]	AR 360° photosphere	Usual training	Orientation in a new clinical environment	Anxiety	−62.55%	*p* = 0.300
Kurt Y (2021) [84]	MAR (Mobile Augmented Reality)	Traditional teaching methods	SC, IM, and IV injections	Skill levels of nursing students	SC median (exp = 23; control = 12) IM median (exp = 31.75; control = 14) IV median (exp = 34.25; control = 22)	*p* < 0.001 *p* < 0.001 *p* < 0.001
McCafferty, K (2022) [19]	AR posters integrated into a flipped classroom model	Traditional, faculty-led paper case studies in a flipped classroom model	A case of COPD exacerbation in a 75-year-old man (pathophysiology, pharmacology, and health assessment.)	Knowledge acquisition Student satisfaction	Not available (similar results in intervention and control group)	NA
Rodríguez-Abad C (2022) [34]	AR	Traditional teaching methods	Leg ulcer care	Knowledge and skills	Knowledge and skills = 25.08%	*p* < 0.001
Uymaz P (2022) [85]	AR (AsthiAR and SnapLearn)	No	Anatomy	Behavioral intention to use AR Use behavior of AR AR acceptance	Not applicable	NA
Wunder L (2020) [86]	ARH (Magic Leap One™)	No	Emergent fire during a simulated tracheostomy procedure in a Mixed Reality operating room environment	Technical skills; Non-technical skills	Not applicable	NA
Zhou G (2022) [87]	AR 3D projection system overlaying dynamic facial expressions onto a patient mannequin	traditional mannequin	stroke simulation scenario	Gaze behavior Simulation performance Learning experience	Time critical phase = −53.19% Simulation performance = 12.5%	*p* < 0.05 *p* < 0.05
Mixed Reality/Holographic Simulations						
Barnett T (2017) [88]	Collaborative “Helping Hands” tele-assistance system (Mixed Reality)	No	Performing a simple wound dressing on a mannequin	Usability ratings from instructors and students, workload assessment.	Not applicable	NA
Hauze S (2019) [89]	holographic MR simulation	Traditional mannequin-based simulation or standard lectures	Evaluating students’ motivation to learn through holographic MR simulations (anaphylaxis)	Student motivation to learn, student satisfaction, and self-confidence Perceived educational value Knowledge of anaphylaxis	NA	NA
Frost J (2020) [90]	MR technology Holopatient	No	Hologram patient anaphylaxis	Students’ learning experience using the HoloLens Impressions and feelings	Not applicable	NA
Frost J (2020) [91]	MR assesses the perceptions of patient needs through a holographic patient (Holopatient)	No	MR holographic patient with symptoms of a myocardial infarction	Directed content analysis	Not applicable	
Son Y (2023) [92]	MR Holopatient	No	Care of COVID-19 patient	Benefits Barriers Learning motivation Critical thinking skills Self-confidence Knowledge acquisition	Not applicable	NA
Mixed Reality/Holographic Simulations						
Lee Y (2020) [93]	IVR 360-degree videos and HMDs AR headset (Microsoft HoloLens)	No	Care of patients with schizophrenia in a psychiatric ward environment	ease of use and usefulness advantages and challenges	Not applicable	NA
Plotzky C (2023) [94]	VR with high or low fidelity Compared educational outcomes achieved by three groups learning with either two different simulation variants or video training on endotracheal suctioning	Video tutorial Two groups of VR simulation against one group of video training	Endotracheal suctioning (ETS)	Knowledge acquisition ETS Skill performance Learner satisfaction Technology acceptance	Knowledge acquisition NA Skill demonstration Video/VR high: Cohen’s *d* = 1.15 Video/VR low: Cohen’s *d* = 0.83 Satisfaction: VR/Video low: Cohen’s *d* = 0.70	*p* = 0.730 *p* < 0.001 *p* < 0.001 *p* = 0.004
Kim SK (2023) [95]	Mixed simulation (360° Virtual Reality and a high-fidelity simulator)	case discussions based on written scenarios	Care for patients with arrhythmia	Decision-making anxiety and confidence	knowing and acting: 15.07%; seeking information from instructors = 10.30% Anxiety about using resources to gather information = −19.61%	*p* = 0.025 *p* = 0.049 *p* = 0.031
Lam VSF (2020) [96]	Mixed Simulation Model Simulation Ward; two high- and one mid-fidelity patient simulators	No	Case management ability, prioritizing nursing actions, and teamwork communication skills	Clinical competence assessed by QSEN Competency Checklist	knowledge (mean difference = 1.33) skills (mean difference = 1.06) attitudes (mean difference = 1.21)	*p* < 0.05 *p* < 0.05 *p* < 0.05
Liang C (2021) [97]	AR headset (Microsoft HoloLens) projects 3D-animated facial expression onto computerized mannequin for early signs of stroke	No	Identifying and managing early stroke symptoms (drooping, garbled speech) in a clinical setting	Identification of stroke symptoms; FAST assessment	Not applicable	NA
Serious Games/MUVEs						
Rim D (2022) [98]	MUVEs	No	Pediatric nursing (hypoglycemia, apnea, transfusion, dehydration, home-visiting scenarios)	Clinical judgment Nursing competency	Clinical judgment = 24.02% Nursing competency = 8.37%	*p* < 0.001 *p* < 0.001
Other/Conceptual/Not classified						
Curro-Urbano O (2022) [99]	VS as a teaching strategy	No	Nursing education during the COVID-19 pandemic	Student satisfaction Academic performance	Satisfaction and academic performance = −1.3387.	*p* = 0.10
Zieber M (2018) [100]	High-fidelity immersive simulation with props (high-fidelity mannequins) and a practical component (high-fidelity simulators capable of replicating cardiac emergencies, ECG)	No	Advanced cardiac skills	Competence Confidence Knowledge retention	NA NA NA	*p* = 0.007 *p* < 0.001 *p* < 0.001

AI = Artificial Intelligence; AR = Augmented Reality; ARH = Augmented Reality headset; BLS = Basic Life Support; IVR= Immersive Virtual Reality; CPR = cardiopulmonary resuscitation; COPD = Chronic Obstructive Pulmonary Disease; HMDs = head-mounted displays; MUVEs = Multi-User Virtual Environments; VRS = Virtual Reality Simulation; ER = Emergency Room; ETS = endotracheal suctioning; NASC-CDM© = Nursing Anxiety and Self-Confidence with Clinical Decision-Making Scale; ^†^ differences pre–post; SDOH = social determinants of health; QSEN = Quality and Safety Education for Nurses Competency Checklist; SUS = System Usability Scale; NA = not applicable/not available; FAST = facial drooping, arm weakness, speech difficulties, time; PIE-SES = Patient Clinical Information Exchange and Interprofessional Communication Self-Efficacy Scale; participants’ perceptions using the Technology Acceptance Model (TAM) and Agent Persona Instrument (API) scales; NA = Not applicable/Not available; VS = Virtual Simulation.

**Table 3:** Categorization of included studies by type of intervention.

Intervention Type	Studies	Number of Studies	%
Immersive Virtual Reality/Virtual Simulation	[22,72,73,74,75,76,77,78,79,80,81],	11	33.3%
Augmented Reality	[8,19,34,82,83,84,85,86,87],	9	27.3%
Mixed Reality/Holographic Simulations	[88,89,90,91,92],	5	15.2%
Mixed Simulations (360°/High-Fidelity/Blended)	[93,94,95,96,97],	5	15.2%
Serious Games/MUVEs	[98]	1	3.0%
Other/Conceptual/Not classified	[99,100]	2	6.1%

MUVEs = Multi-User Virtual Environments.

**Table 4:** Newcastle–Ottawa Quality Assessment Scale cohort studies (number of stars ✸).

Study		Selection			Comparability		Exposure		Final Score
	Representativeness of the Exposed Cohort	Selection of the Non-Exposed Cohort	Ascertainment of Exposure	Demonstration that the Current Outcome Was Not Present at the Start of the Study	Comparability of Cohorts Based on Design or Analysis	Assessment of Outcome	Was Follow-Up Long Enough for Outcomes to Occur	Adequacy of Follow-Up of Cohorts	
	1	2	3	4	5	6	7	8	✸
Adhikari R (2021) [72]			✸	✸	✸		✸	✸	5
Ball S (2020) [83]			✸	✸	✸✸			✸	5
Flo J (2021) [74]				✸	✸			✸	3
Hanson J (2019) [75]			✸	✸	✸				3
Hoffman JL (2023) [76]			✸	✸	✸				3
Lau ST (2023) [77]			✸	✸	✸			✸	4
Liaw S (2023) [79]			✸	✸	✸			✸	4
Rim D (2022) [98]			✸	✸	✸			✸	4
Vaughn J (2016) [8]			✸	✸	✸			✸	4
Wunder L (2020) [86]			✸	✸	✸	✸		✸	5
Zhou G (2022) [87]			✸		✸	✸		✸	4
Zieber M (2018) [100]			✸	✸	✸	✸		✸	5

**Table 5:** Newcastle–Ottawa Quality Assessment Scale cross-sectional studies.

Study	Selection				Comparability	Outcome		Final Score
Reference	Representativeness of the Sample	Sample Size	Non-Respondents	Ascertainment of the Exposure (Risk Factor)	Outcome Groups Comparable Confounding Factors Controlled	Assessment of Outcome	Statistical Test	Total
Barnett T (2017) [88]					✸		✸✸	3
Cieslowski B (2023) [73]					✸	✸	✸✸	4
Curro-Urbano O (2022) [99]				✸	✸		✸	3
Foronda CL (2018) [22]					✸		✸	2
Frost J (2020) [90]					✸		✸	2
Lam VSF (2020) [96]				✸		✸	✸	3
Lau ST (2023) [78]				✸			✸	2
Lee Y (2020) [93]					✸		✸	2
Liang C (2021) [97]					✸		✸	2
Uymaz P (2022) [85]					✸		✸	2

**Table 6:** Evaluation of the quality of the studies using the critical appraisal skills program (CASP) qualitative studies checklist (n  =  23).

Author (Year)	Philosophical Congruity	Objective Congruity	Method Congruity	Analysis Congruity	Interpretation Congruity	Researcher Position	Researcher Influence	Participant Voices	Ethical Considerations	Conclusions and Analysis:	Global
Adhikari R (2021) [72]	1	1	1	1	1	UC	UC	1	1	1	High
Flo J (2021) [74]	1	1	1	1	1	UC	UC	1	1	1	High
Foronda CL (2018) [22].	1	1	1	1	1	UC	0	1	1	1	High
Frost J (2020) [91]	UC	1	1	1	1	0	0	1	1	1	Moderate
Hoffman JL (2023) [76]	1	1	1	1	1	0	0	1	1	1	High
Lau ST (2023) [77]	UC	1	1	1	1	0	1	1	1	1	High
Lau ST (2023) [78]	1	1	1	1	1	1	0	1	1	1	High
Lee Y (2020) [93]	1	1	1	1	1	UC	0	1	1	1	High
Liang C (2021) [97]	0	1	1	1	1	0	UC	1	1	1	High
Liaw S (2023) [79]	UC	1	1	1	1	UC	0	1	1	1	Moderate
Plotzky C (2023) [94]	UC	1	1	1	1	UC	UC	1	1	1	Moderate
Rim D (2022) [98]	1	1	1	1	1	1	UC	1	1	1	High
Rushton M (2020) [81]	1	1	1	1	1	UC	UC	1	1	1	High
Son Y (2023) [92]	1	1	1	1	1	0	UC	1	1	1	High
Zhou G (2022) [87]	UC	1	1	1	1	0	0	1	1	1	Moderate
Zieber M (2018) [100]	1	1	1	1	1	0	UC	1	1	1	High

1 = Yes; 0 = No; UC (unclear or cannot tell); NA (not applicable). High quality: studies that meet most or all of the appraisal criteria (8–10 “Yes” responses). Medium quality: studies that meet some appraisal criteria (5–7 “Yes” responses). Low quality: studies that meet few of the appraisal criteria (0–4 “Yes” responses).

**Table 7:** Summary of meta-analyses of educational outcomes in MR-based nursing education.

Outcome	Study Type	N	Model	Effect Size (Cohen’s d)	95% CI	I^2^ (%)	Egger Intercept ^1^	95% CI
Knowledge	Controlled	5	RE	0.093	−0.16, 0.36	0.47	0	−2.36–2.36
Skills	Controlled	10	RE	−0.50	−0.54, 0.44	0.89	−0.67	−2.67–1.33
Anxiety ^1^	Controlled	2	FE	−0.73 **	−1.15, −0.31	-	-	-
Trust	Controlled	4	RE	0.93	−1.44, 3.30	0.98	−2.85	−14.12–8.40
Knowledge	Uncontrolled	5	RE	0.65 *	0.22, 1.08	0.73	0.63 *	−0.09–1.37

^1^ Egger’s Regression-Based Test cannot be computed when the number of studies is less than or equal to two. * *p* < 0.01 ** *p* < 0.001. RE = random effects; FE = fixed effects.

## Data Availability

The data are included in the Supplementary Materials.

## Supplementary Materials

The following supporting information can be downloaded at https://www.mdpi.com/article/10.3390/nursrep15050137/s1, Figure S1: JoinPoint analysis of the number of papers on Mixed Reality; Figure S2: Funnel plot with respect to the meta-analysis of anxiety; Figure S3: Funnel plot for trust; Figure S4: Funnel plot for skills; Figure S5: Funnel plot with respect to the meta-analysis of knowledge (uncontrolled studies); Figure S6: Funnel plot with respect to the meta-analysis of knowledge (controlled studies); Table S1: JoinPoint analysis of the number of papers on Mixed Reality; Data_file.xlsx: File containing the data.

## Public Involvement Statement

There was no public involvement in this research.

## Guidelines and Standards Statement

This manuscript was drafted against PRISMA (the full name of reporting guidelines and citations) for systematic review research.

## Abbreviations

The following abbreviations are used in this manuscript:

MDPI	Multidisciplinary Digital Publishing Institute
AR	Augmented Reality
AI	Artificial Intelligence
IS	immersive simulation
MR	Mixed Reality
MUVEs	Multi-User Virtual Environments
VR	Virtual Reality
WOS	Web of Science
XR	Extended Reality
