# Supplementary material for: Mixed Reality in Undergraduate Nursing Education: A Systematic Review and Meta-Analysis of Benefits and Challenges
Source: Nurs Rep. 2025 Apr 22;15(5):137. doi: 10.3390/nursrep15050137 (PMC12113701; doi:10.3390/nursrep15050137)
Supplement: Supplementary file 1 [file nursrep-15-00137-s001.zip › Virtual_Reality_Suplementary - proof_corrected.pdf]

Table S1. Joinpoint analysis for the number of papers on mixed reality.

Periods	Years	APC (95% CI)	*p*
Total Period	2016–2023	37.8 (26.6; 49.9)	<0.001
Period 1	2016–2020	56.7 (48.0;71.5)	0.001
Period 2	2020–2023	13.0 (-12.0;26.7)	0.262

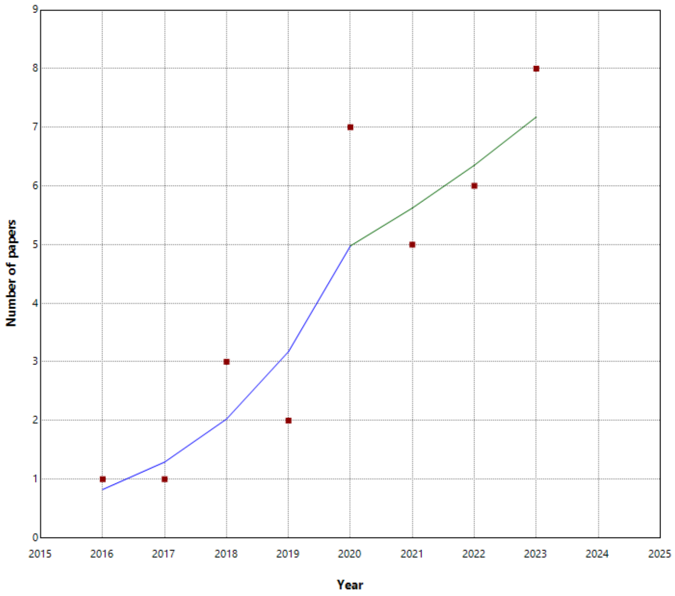

Figure S1. Trends in the number of papers on mixed reality (2016–2023) indicating joinpoints.

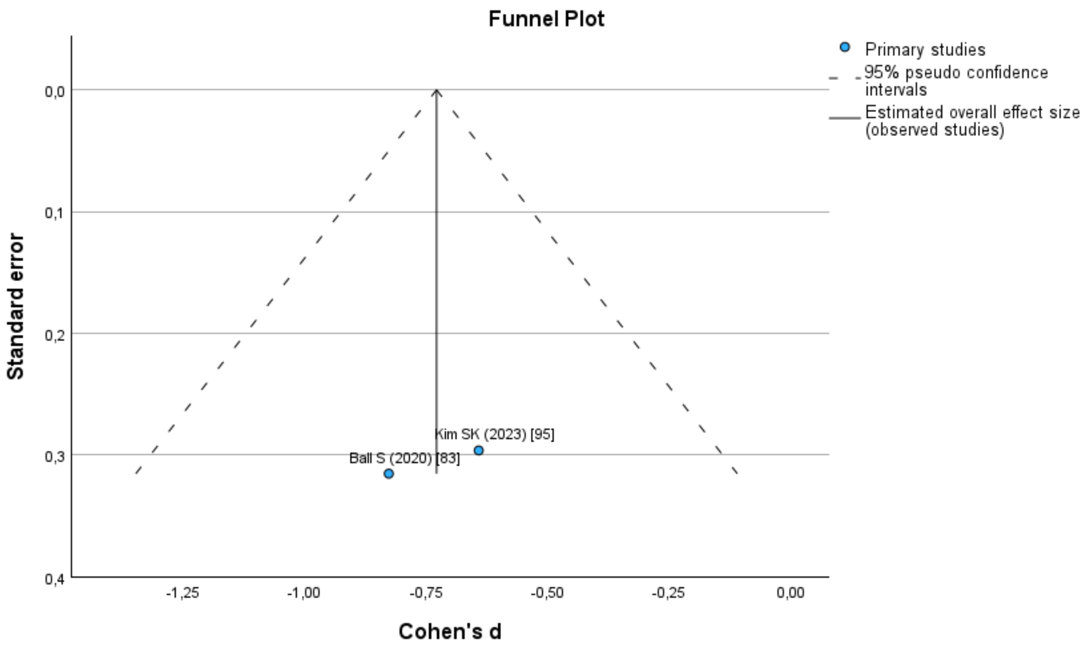

Figure S2. Funnel plot of the meta-analysis of anxiety.

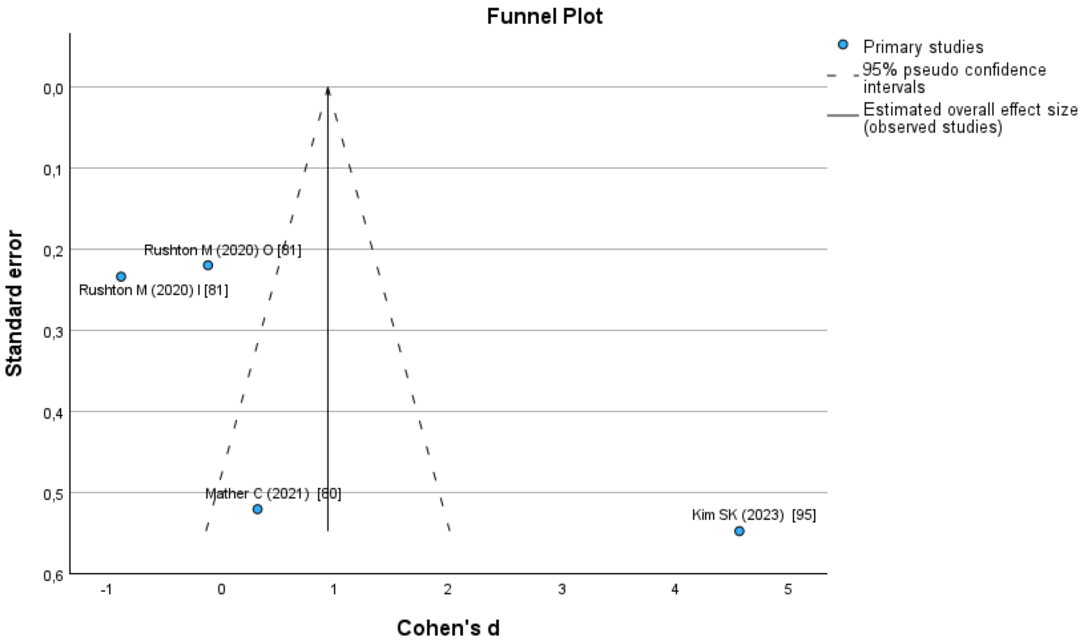

Figure S3. Funnel plot of the meta-analysis of Trust.

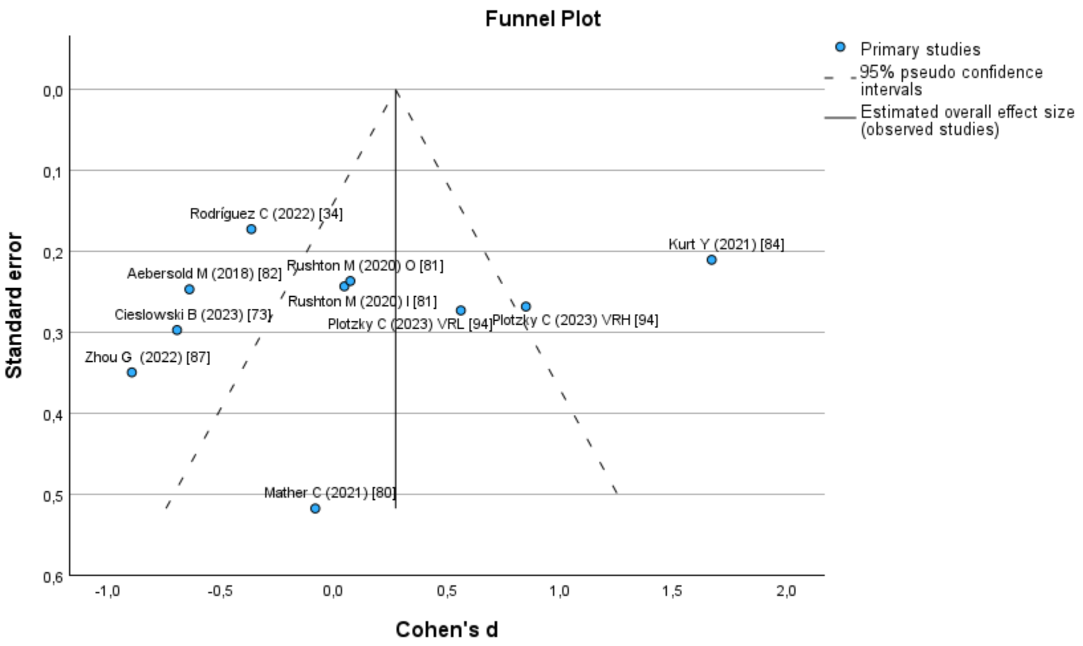

Figure S4. Funnel plot of the meta-analysis of Skills.

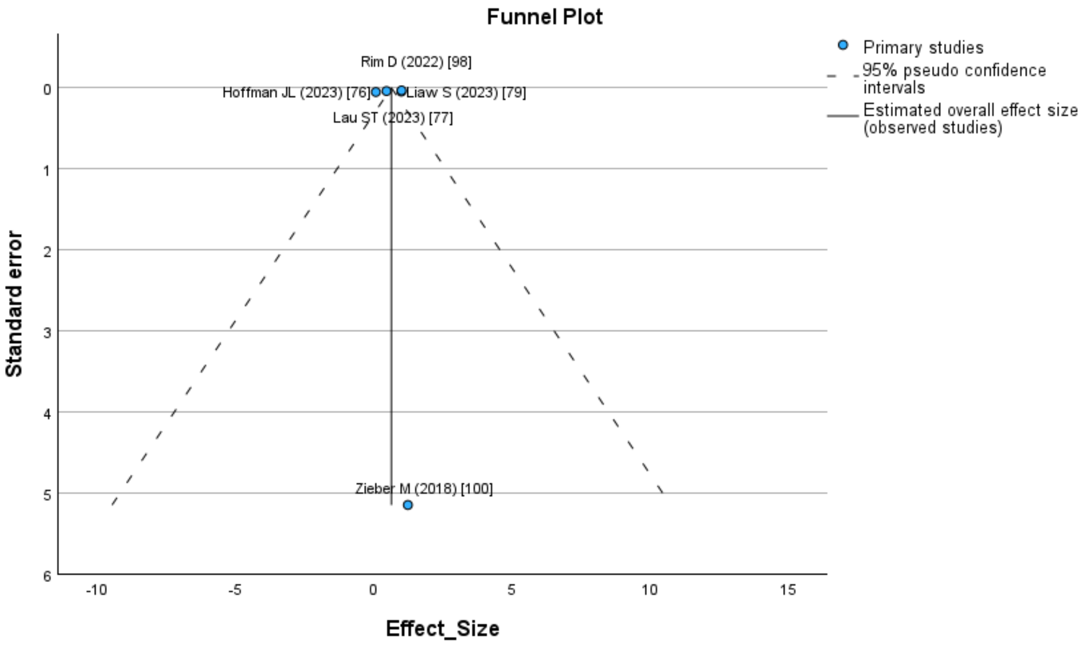

Figure S5. Funnel plot of the meta-analysis of knowledge (uncontrolled studies).

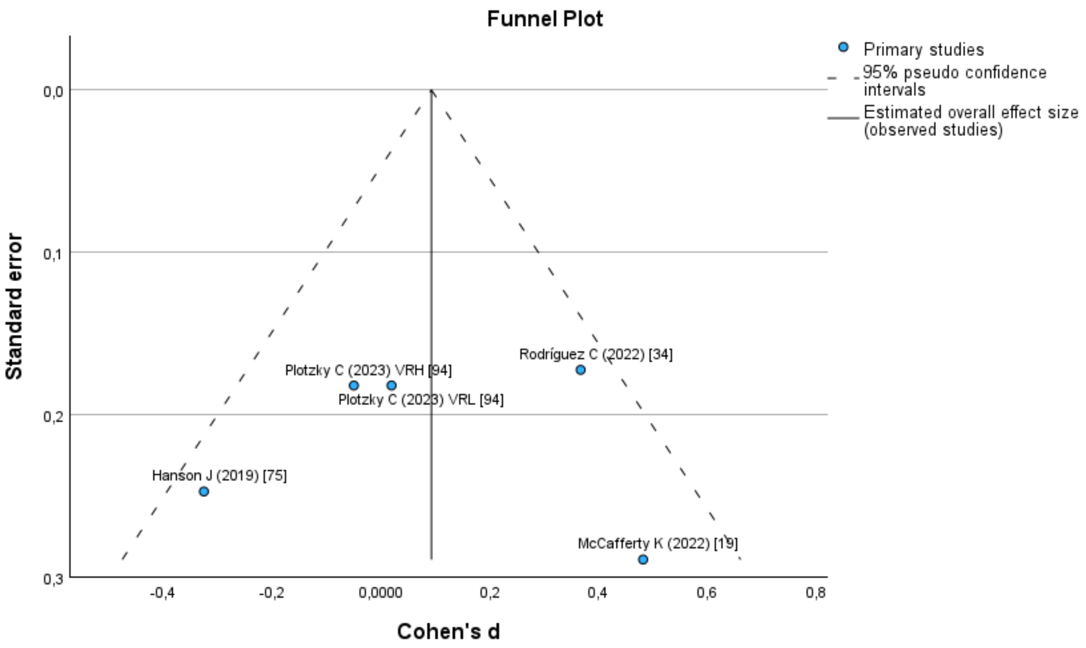

Figure S6. Funnel plot of the meta-analysis of knowledge (controlled studies).
